# PCR-Based Detection and Genetic Characterization of Parainfluenza Virus 5 Detected in Pigs in Korea from 2016 to 2018

**DOI:** 10.3390/vetsci10070414

**Published:** 2023-06-25

**Authors:** Ha-Thai Truong, Van-Giap Nguyen, Le-Bich-Hang Pham, Thi-My-Le Huynh, Jasper Lee, Su-Jin Hwang, Jae-Myun Lee, Hee-Chun Chung

**Affiliations:** 1 Department of Veterinary Microbiology and Infectious Diseases, Faculty of Veterinary Medicine, Vietnam National University of Agriculture (VNUA), Hanoi 100000, Vietnam; ththai@vnua.edu.vn (H.-T.T.); nvgiap@vnua.edu.vn (V.-G.N.); huynhtmle@vnua.edu.vn (T.-M.-L.H.); 2 Institute of Genome Research, Vietnam Academy of Science and Technology, 18 Hoang Quoc Viet, Cau Giay, Hanoi 100000, Vietnam; plbhang@igr.ac.vn; 3 Computational Neurobiology Laboratory, Salk Institute of Biological Sciences, La Jolla, CA 92037, USA; tigerjasper122@gmail.com; 4 Department of Microbiology and Immunology, Institute for Immunology and Immunological Diseases, Brain Korea 21 Project for Medical Science, Yonsei University College of Medicine, Seoul 03722, Republic of Korea; hapisujin@yuhs.ac (S.-J.H.); jaemyun@yuhs.ac (J.-M.L.); 5 Department of Microbiology and Immunology, Institute for Immunology and Immunological Diseases, Yonsei University College of Medicine, Seoul 03722, Republic of Korea

**Keywords:** porcine parainfluenza virus 5, detection, genetic analysis, Republic of Korea

## Abstract

**Simple Summary:**

Parainfluenza virus 5 (PIV5) has been known for a long time. The virus is detected in various animals suffering from different clinical diseases. However, the pathogenicity of PIV5 is largely unconfirmed. Thus, it is necessary to continue researching for the prevalence, genetic diversity, and evolution characteristic of PIV5. The results of this study confirmed a permanent prevalence of PIV5 in Republic of Korea, identified three field strains with more divergence, and elucidated the evolutionary path of PIV5 in which a few codons of the F and HN genes had an elevated rate of non-synonymous substitutions.

**Abstract:**

This study applied a molecular-based method to detect parainfluenza virus 5 (PIV5) collected from 2016 to 2018 in nine provinces of Republic of Korea. We demonstrated that PIV5 was detectable in both serum and pooled organs at an average positive rate of 1.78% (99/5566). Among these, the complete genome sequence of 15,246 nucleotides was obtained for 12 field strains. Three out of the 12 strains had the lowest genetic identity (96.20–96.68%) among the 21 porcine PIV5 genomes collected in Germany, China, India, and Republic of Korea from 1998 to 2017. By analyzing a large collection of complete genome sequences of the structural protein-coding F and HN genes, this study proposed a classification of PIV5 into two lineages, 1 and 2, and identified that group 2.2.2 within sub-lineage 2.2 was substantially divergent. The evolution of two structural protein-coding genes was largely under purifying selection. A few codons (6/9 for the F gene, 7/8 for the HN gene) had elevated dN/dS values, which were loaded on internal branches and were predicted to be related to beneficial trait(s) of the virus.

## 1. Introduction

Parainfluenza virus 5 (PIV5) is an enveloped, negative-sense, single-stranded RNA virus of the genus *Orthorubulavirus*, subfamily *Rubulavirinae*, family *Paramyxoviridae* [1]. The genome of PIV5 is 15,246 nucleotides, which encode eight proteins in the order of nucleocapsid protein (NP), V protein (V), phosphoprotein (P), matrix protein (M), fusion protein (F), small hydrophobic integral membrane protein (SH), hemagglutinin-neuraminidase (HN), and the polymerase protein (L) [2,3,4]. For some strains of PIV5 (SER, KNU-11), the SH protein is not expressed [5,6]. Two glycoproteins, F and HN, are on the viral surface. The HN protein is responsible for binding to sialic acid-containing cellular molecules and for enzymatic cleavage of the sialoconjugate, while the F protein is involved in envelope-to-cell and cell-to-cell fusion [7]. Of the significant veterinary paramyxoviruses, such as the Newcastle disease virus, the two structure proteins mentioned above are major determinants for virulence and immunity [8]. However, little is known about PIV5 [9].

To date, PIV5 has been reported worldwide in a wide host range, such as humans [6], pigs, dogs, cows, lesser panda, and tiger [10,11,12,13,14]. Regardless of the infected hosts, PIV5 has low genetic diversity and is phylogenetically indistinguishable [6,15]. PIV5 is also found to infect a wide range of mammalian cell types and possesses a risk for potential zoonosis [16]. Although PIV5 is normally detected and/or isolated from cases with various clinical signs, the pathogenic role of PIV5 in most species (except dogs) has not been confirmed. In two recent publications, one pointed out the possibility of genetic diversity affecting the pathogenicity of the parainfluenza virus [17], and the other suggested the importance of continuing research in identifying the genetic diversity of the parainfluenza virus [18]. As a result, this study attempted to provide data on PIV5 infection in pigs in Republic of Korea based on a large-scale nationwide screening. We then applied bioinformatic tools to analyze the molecular evolution of PIV5 on the two main structural protein-coding genes (F and HN genes).

## 2. Materials and Methods

### 2.1. Screening for PIV5 in Pigs

Between January 2016 and December 2018, there were a total of 5566 samples (Table 1) collected from 213 domestic farms in nine Korean provinces that were sent to our laboratory for investigation of common pig pathogens. The samples collected for this study were classified into two groups: serum (n = 4590) and pooled organs (n = 976). The pooled internal organs (lymph nodes, heart, lungs, liver, spleen, and kidneys, but not intestines) were from dead pigs. Meanwhile, the serum was submitted for herd monitoring of PRRSV and PCV2. There were no cases where both serum and pooled organs were tested from the same animal in this study.

The total nucleic acid was extracted using a Viral DNA/RNA Extraction Kit (Introns Biotechnology Inc., Gyeonggi, Republic of Korea). The extracted nucleic acid was then converted into cDNA with the use of random hexamers and a commercial RNA to cDNA EcoDry Premix kit (Clontech, Otsu, Japan) following the manufacturer’s protocol. The presence of PIV5 was detected with in-house specific primers based on the sequence of the KNU-11 strain (KC852177) (Supplementary Table S1).

### 2.2. Complete Genome Sequencing of PIV5

Among the PIV5 positive samples, 12 strains (T220, T263, T361, T398, T399, T434, M32, M129, M197, M293, N99, and N163) were randomly selected and completely sequenced with a primer-walking method with 15 pairs of specific primers (Supplementary Table S1). The specific PCR products were purified with gel extraction and further processed for TA cloning and transformation. The Sanger sequencing was performed by Macrogen Inc. (Seoul, Republic of Korea). The full-length genomes of 12 PIV5 strains were registered in GenBank (accession numbers MK423232 to MK423243).

### 2.3. Genetic Characterization and Phylogenetic Classification of PIV5

For the genetic analysis, this study collected three different datasets: complete genome (n = 55), F gene (n = 96), and HN gene (n = 72). The number of sequences between the datasets was different due to the lack of either the F or HN gene for the same strain. The detailed information of the sequences used in this study are given in Supplementary Table S1.

For the complete genome comparison of PIV5 detected from pigs (n = 24), SDTv1.2 [19] was used to compute the pairwise sequence identities based on multiple sequence alignments. The input options were Muscle as an alignment program and clustering sequences with a neighbor joining tree. Additionally, a sliding window analysis implemented in SimPlot [20] was used to plot the similarity versus position among the porcine PIV5 genomes. The query sequence was selected as the SER strain (JQ743328), which was collected from an aborted pig fetus in 1998.

For the phylogenetic classification, phylogenetic trees based on the complete genome, F gene, and HN gene were inferred with the maximum likelihood method implemented in IQ-TREE v1.6.12 [21]. The best nucleotide substitution model for each dataset was selected automatically by specifying the ‘-m MFP’ option. The branch supports of the phylogenetic trees were calculated via BOOSTER [22].

For the proposal of a new phylogenetic group, an additional analysis of the pairwise genetic distances (p-distances) was performed. In brief, the p-distance for each dataset (indicated in Supplementary Table S1) was calculated using MEGA11 software [23]. The option for gap treatment was specified as “partial deletion”. Microsoft Excel (Microsoft 365 program) was used to draw the frequency distribution histogram of the p-distance.

### 2.4. Evolutionary Analyses of PIV5

The M0 and M8 models in the Codeml program (PAML 4.8) [24] were applied for the codon-based alignment of the complete F and HN genes of PIV5. The input phylogenetic tree for the codon-based analysis was that inferred with the IQ-TREE program (Section 2.3). The dN/dS values estimated for the entire F and HN genes or for each codon position were obtained from the M0 and M8 models, respectively. A bioinformatic tool (Treesub, https://github.com/tamuri/treesub, last accessed 10 April 2023) was used to annotate the ancestral non-synonymous changes along the internal branches of the phylogenetic trees. Those changes were inferred under the M8 model of the Codeml program [24]. The internal branches (branches connecting two nodes) and external branches (branches connected to leaves) were defined according to a previous publication [25].

## 3. Results

### 3.1. Detection of PIV5 in Pigs in Korea from 2016 to 2018

**Figure 1:**
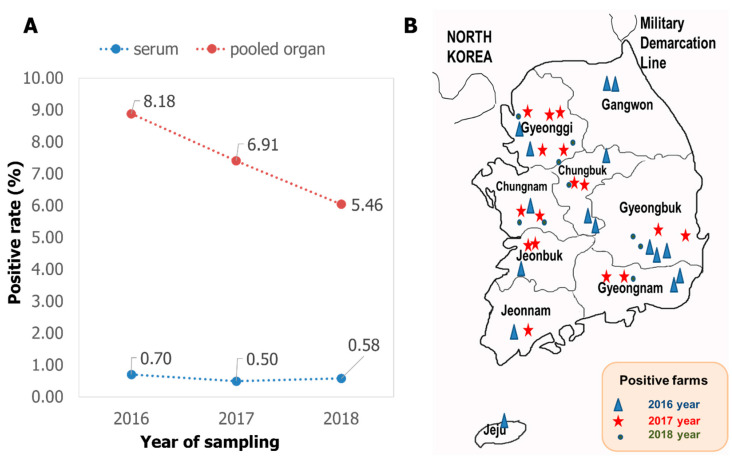
Summary of the prevalence of PIV5 in pigs in Korea. (**A**) The positive rate for serum and pooled organs from three consecutive years. (**B**) The chronological and geographic distribution of positive farms.

The screening results with RT-PCR carried out on the total 5566 samples showed that PIV5 was present in Korean domestic swine herds with an average positive rate of 1.78% (99/5566). Looking at the type of testing material, serum was consistently less than 1% positive, while the pooled internal organs were always at least ten times higher (Figure 1A). Looking at the geographic distribution, PIV5 was detected in all nine provinces of Republic of Korea, of which the two main swine raising areas (Gyeonggi and Gyeongbuk) had the virus detected during all three years of the investigation.

### 3.2. Genomic Comparison of PIV5 Detected in Pigs in Korea from 2016 to 2018

**Figure 2:**
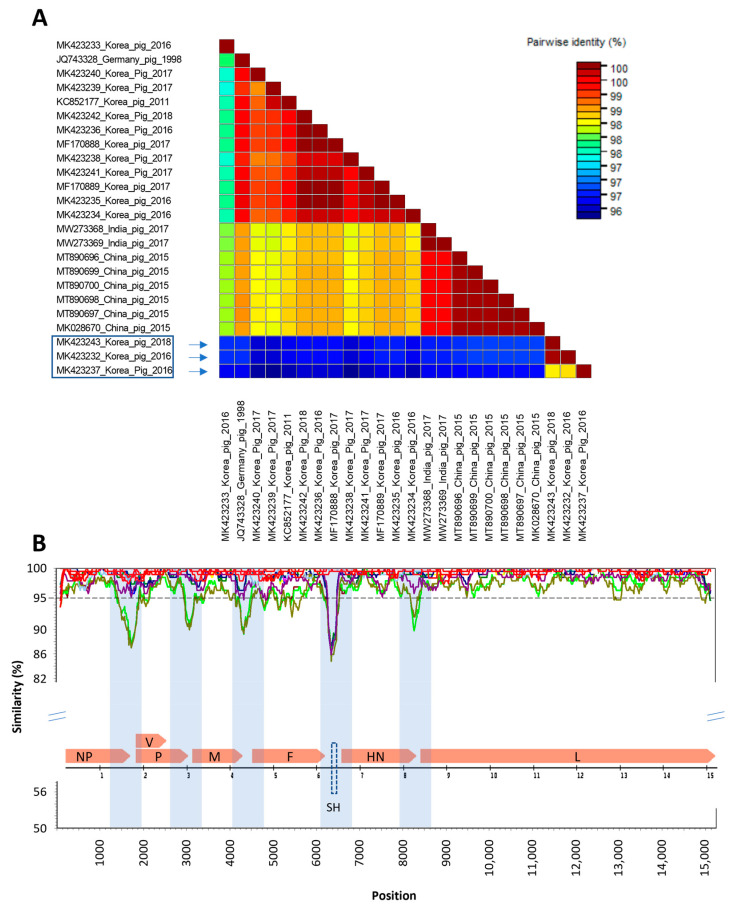
Genome comparison of Korean porcine PIV5. (**A**) Pairwise comparison between 24 porcine PIV5 genomes. Box indicated porcine PIV5 strains had the lowest genetic identity to the rest of the 21 porcine PIV5 genomes. (**B**) The similarity sliding window analysis showing genome-wide comparison. The dashed line indicates the genetic similarity of 95%. Genetic regions having significant drops of similarity are highlighted. The insert is the representation of the SER genome (JQ743328) with the location of the seven genes flanked by the intergenic regions. The prediction of the SH protein-coding gene is shown with a dashed box.

A total of 24 complete genomes of PIV5 detected from pigs with clear information are available in GenBank, all of them with 15,246 nucleotides. This study analyzed the genomic diversity of Korean porcine PIV5 in the context of all available PIV5 detected in pigs in some other countries. Using the genome of SER (the earliest porcine sequence) as the query, the genetic similarity between the other porcine PIV5 genomes were analyzed (Figure 2).

The results of the pairwise comparison (Figure 2A) revealed that, while some of the Korean porcine PIV5 strains identified in this study had clustered together with previously characterized porcine PIV5 strains, the three Korean porcine PIV5 strains collected in 2016 and 2018 (boxed) had the lowest genetic identities (96.20–96.68%) compared to the other 21 porcine PIV5 genomes collected in Germany, China, India, and Republic of Republic of Korea from 1998 to 2017. The other 12 Korean porcine PIV5 sequences showed genetic similarity above 97.70% to the strains collected in the other countries. Along the genome of porcine PIV5, there were at least five genetic regions that had significant drops of similarity (below 95%). Interestingly, all of these regions were located in the intergenic regions (shades, Figure 2B). Among the seven protein-coding genes of 22 porcine PIV5 strains (except for the SH gene that is absent in some strains), the F gene had the lowest nucleotide similarity (more regions below the dashed line of 95% identity).

### 3.3. The Genetic Classification of PIV5

After the 2019 publication of the most divergent PIV5 strain collected in Russia (MK593539) [18], there has not been many further studies on the genetic evolution of porcine PIV5 in particular and PIV5 in general to our knowledge. With 12 complete genome sequences generated in this study, it is our interest to elucidate the genetic relationships of the porcine PIV5 strains in a broad context with the PIV5 strains from different species based on the genomic sequence and the two main structural protein-coding F and HN genes.

**Figure 3:**
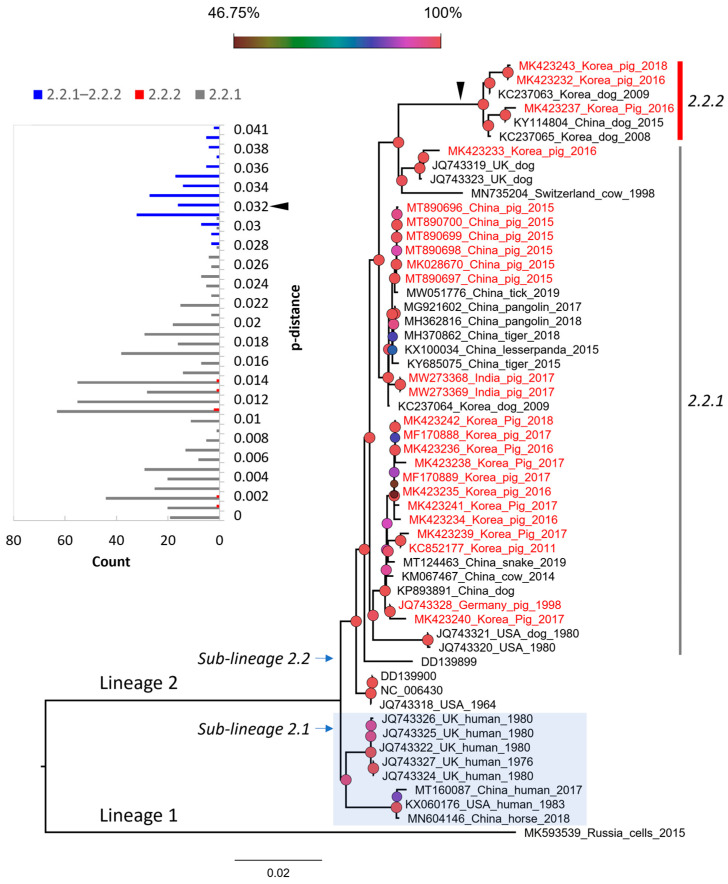
The maximum likelihood phylogenetic tree of PIV5 based on the genomic sequences. The tree is midpoint rooted. The leaves of the phylogeny are given in the format of GenBank_country_host_year of isolation. Note that some leaves do not have complete information. The sequences from pigs are colored in red. The shaded area indicates the cluster of sequences detected in human and horse. The inserted histogram of the pairwise p-distances that are within (2.2.1 or 2.2.2) and between groups (2.2.1 and 2.2.2). The arrowhead indicates the distance threshold of 0.032 dividing the two groups. The legend represents the bootstrap support value, of which the node shapes are colored and sized accordingly. The scale bar indicates the number of nucleotide substitutions per site.

Based on the complete genome, PIV5 was divided into two proposed lineages (this study). Lineage 1 contained a single strain (MK593539), which is the most divergent PIV5 known to date. Lineage 2 contained sub-lineage 2.1 of the cluster of sequences detected in human and horse, and sub-lineage 2.2 of the mixture sequences detected in various animals, such as pig, dog, cow, tiger, etc. Based on the pattern of branching, sub-lineage 2.2 could be further divided into two groups, namely 2.2.1 and 2.2.2. Of the two, group 2.2.2 showed substantially more divergence than the rest. This classification was also supported by a bimodal distribution and marginal overlap of the genetic distance between the groups (inserted histogram, Figure 3). All porcine PIV5 collected in different countries (China, India, Republic of Korea) (highlighted in red, Figure 3) fell within the two mentioned groups. The Korean porcine PIV5 fell into the two groups of 2.2.1 and 2.2.2.

**Figure 4:**
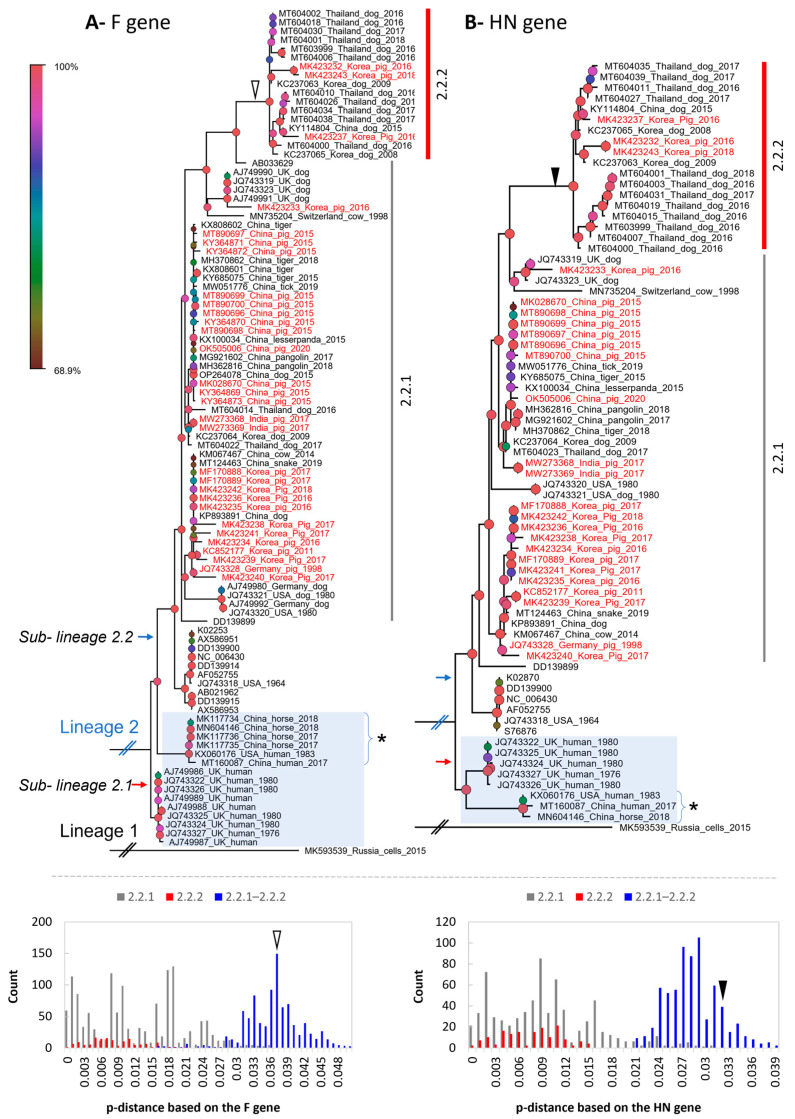
The maximum likelihood phylogenetic tree of PIV5 based on the structure of the F and HN genes. The tree is midpoint rooted. The leaves of the phylogeny are given in the format of GenBank_country_host_year of isolation. Note that some leaves do not have complete information. The number of leaves between the trees are different due to the lack of either the F or HN gene for the same strain. The sequences from pigs are colored in red. The shaded area indicates the cluster of sequences detected in human and horse. The * indicate sequences from human and horse having topological change between the two phylogenetic trees. The inserted histograms indicate the pairwise p-distances based on the F and HN genes within (2.2.1 or 2.2.2) and between the groups (2.2.1 and 2.2.2). Based on the F and HN genes, 0.037 and 0.032 are the respective distance thresholds between the two groups (open and filled arrowheads). The legend bar is the bootstrap support value, of which the node shapes are colored and sized accordingly.

Based on the structure of the F and HN genes (Figure 4), the tree topologies were congruent with that based on the genome in general (Figure 3). A clear separation into two proposed lineages and two sub-lineages was maintained. However, for the F gene-based phylogeny, a topological change was observed in which some sequences from human and horse (*, Figure 4A,B) of sub-lineage 2.1 (based on the genome and HN gene) were now clustered within sub-lineage 2.2. Of porcine PIV5, the two groups of 2.2.1 and 2.2.2 were still observed and were supported by a discrete distribution of p-distances between them (inserted histograms, Figure 4). Group 2.2.2 was more divergent. The Korean porcine PIV5 strains were consistently located in the two groups of 2.2.1 and 2.2.2, as they were on the tree of the complete genome.

### 3.4. The Molecular Evolution of PIV5 Based on Structural Genes

We further studied the evolution of porcine PIV5 on the basis of the two main structural protein-coding genes (F and HN genes) by analyzing (1) the ratio of non-synonymous to synonymous substitution rates (dN/dS) for all codons (sites) in each gene, and (2) the path for which non-synonymous substitutions accumulated along the internal branches of the phylogenetic trees.

**Figure 5:**
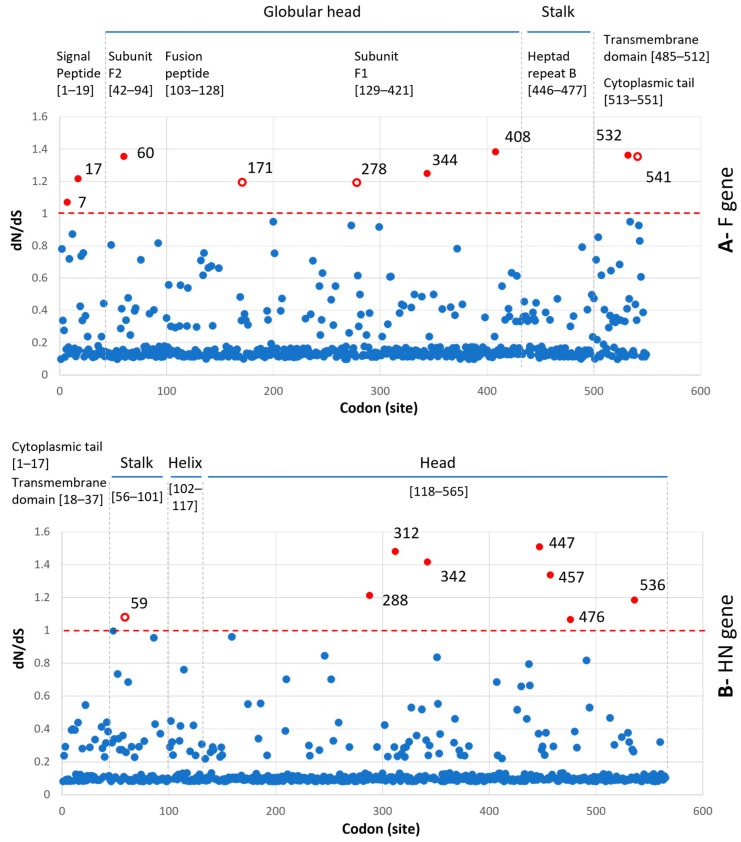
Plot of the dN/dS ratio for the entire (**A**) F gene and (**B**) HN gene. The dN/dS for each codon was estimated under the M8 model. The codons having dN/dS > 1 are highlighted in red. The filled and open red circles are non-synonymous substitutions, which occurred along the internal and external branches of the F- and HN-based phylogenies, respectively. The codons having dN/dS ≤ 1 are highlighted in filled blue circles. Just above each graph is the location (given in square brackets) of the functional regions of the corresponding F [26,27] and HN [28,29] proteins.

**Figure 6:**
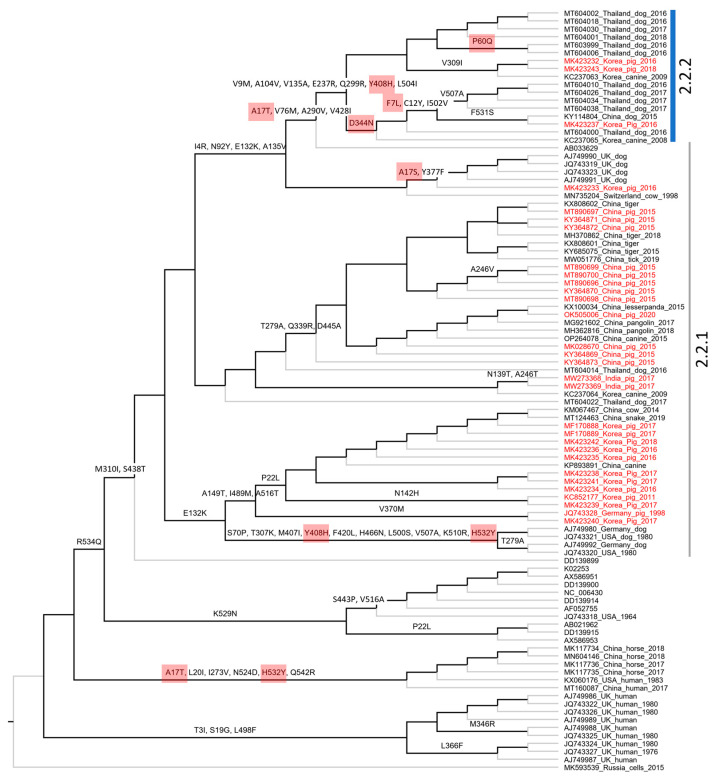
Cladogram showing ancestral non-synonymous substitutions on the internal branches of the F gene-based phylogeny. The non-synonymous codon substitutions are displayed above the branches. The highlighted codons are those that had dN/dS > 1. The codon numbering is according to the first sequence (K02253) of the alignment.

**Figure 7:**
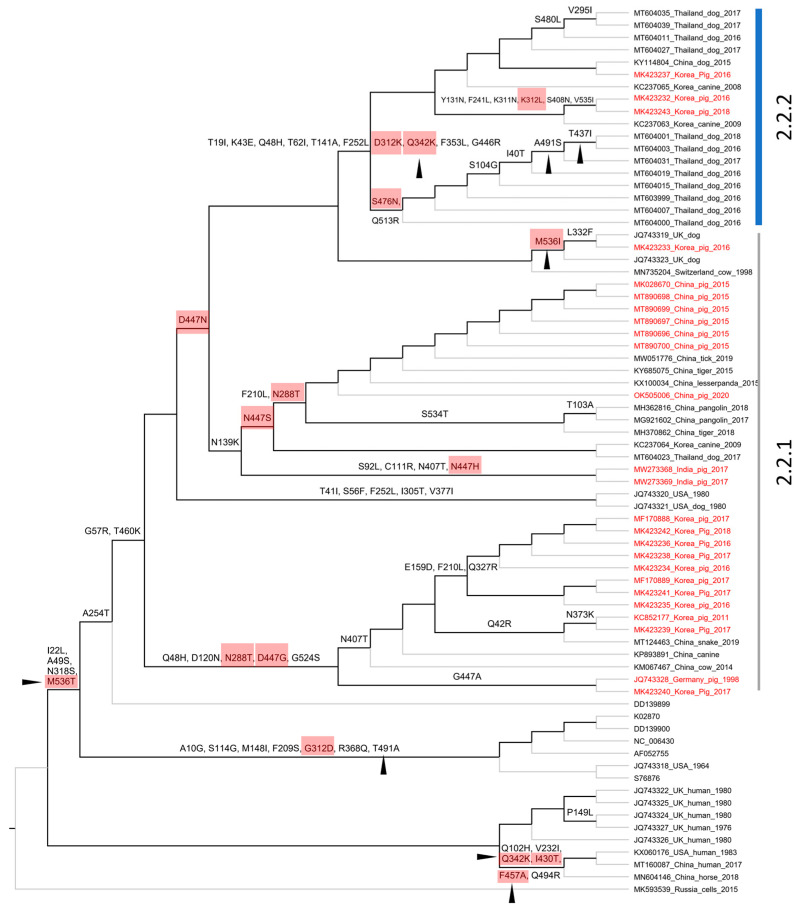
Cladogram showing ancestral non-synonymous substitutions on the internal branches of the HN gene-based phylogeny. The non-synonymous codon substitutions are displayed above the branches. The highlighted codons are those that had dN/dS > 1. The codons related to antibody resistant mutants are indicated with arrowheads. The codon numbering is according to the first sequence (K02870) of the alignment.

Under the M0 model, the dN/dS ratio for the F gene was 0.209 and for the HN gene was 0.154. These results implied that purifying selection (characterized by a higher rate of synonymous substitutions than non-synonymous ones and dN/dS < 1) operates on the F and HN genes of PIV5. At the level of the codon, the estimated dN/dS value for each codon (site) reflected that the majority of codons were under purifying selection (dN/dS < 1, red dashed lines, Figure 5). There were a few codons of the F (9/551) and HN (8/565) genes that had dN/dS > 1. Among the codons, 5/9 and 7/8 codons of the F and HN genes, respectively, were located on the globular head of the encoded proteins. The maps, which show the non-synonymous substitutions along with the internal branches of the F- and HN-based phylogenies, are displayed in Figure 6 and Figure 7.

A total of 67/256 codons for the F gene and 72/565 codons for the HN gene were non-synonymous substitutions occurring along the internal branches (Figure 6 and Figure 7). Based on either the F or HN phylogeny, the current data showed that the number of non-synonymous codons on the internal branches leading to two groups (2.2.1–2.2.2) containing porcine PIV5 was higher than the rest. Additionally, the codons having dN/dS > 1 tended to cluster on the internal branches leading to group 2.2.2 (F-based phylogeny, Figure 6) but were observed to be scattered on the internal branches leading to the two groups 2.2.1–2.2.2 (HN-based phylogeny, Figure 7).

## 4. Discussion

### 4.1. The Detection of PIV5 in Pigs in Korea

In Republic of Korea, PIV5 was reported in dogs with mild pneumonia in 2008–2009 [30], in pigs experiencing respiratory problems in 2011 [5], and in cows associated with neurologic symptoms in 2012 [31]. According to a recent publication, PIV5 was continuously detected in pig samples in 2019 and 2022 [32]. Combined with the results shown in this study covering the period of 2016–2018, an early and permanent prevalence of PIV5 in various animals in Republic of Korea can be confirmed. According to the type of samples, a big difference in the detection rate between serum and pooled organs (Figure 1A) suggested that serum was not a suitable sample for PIV5 detection. A similar recommendation was available in another study, which found that 0 out of 153 sera were PIV5 positive, while two out of 168 lungs were PIV5 positive [32]. During the infection progress, PIV5 induced a rapid, innate, and early adaptive immune response [10]. That might be an explanation for a quick removal of PIV5 and very low detection rate in blood as observed in Figure 1A. For viruses in which the pathogenicity has not yet been confirmed or that depend on unknown factors, it is suggested to detect and compare the positive rate as well as the viral loads between a group of clinically healthy and a group of diseased animals [33,34]. Under this point of view, the current study contained a limitation of having insufficient information to divide the detection rate according to the health status.

### 4.2. The Genetic Characterization and Classification of PIV5 Detected in Pigs

The overall genomic similarity among the porcine PIV5 strains was more than 96% and was congruent with the previous finding of high genetic similarity among PIV5 strains. The observation of higher diversity within the intergenic regions of the PIV5 genome was also in agreement with the previous study [6]. In the previous study, PIV5 strains were mostly clustered based on the natural or laboratory-detected host [6]. In this study, a large dataset of PIV5 sequences was applied for phylogenetic reconstruction, which in turn can systematically evaluate the diversity of PIV5 circulation worldwide. Except for the sub-lineage 2.1 that contained most of the human collected strains, a host diversity of the closed related strains within the remaining groups was observed. This result was in accordance with those reported elsewhere [18]. Additionally, in this study, a more divergent group was initially observed within the sub-lineage 2.2 (Figure 3 and Figure 4).

Despite being isolated from sick animals showing various clinical signs [15,16,31,35,36], PIV5 in general and PIV5 isolated in pigs in particular have not been confirmed for their pathogenic roles. Some studies relied on a specific isolate [10,37] and did not find evidence to support that PIV5 induced significant illness in infected animals. On the other hand, a recent infection experiment suggested that genetic differences between strains of porcine parainfluenza virus 1 may impact clinical outcomes [17]. With the identification of a higher diversity PIV5 in group 2.2.2 (Figure 3 and Figure 4), it is worth pursuing further experimental studies using the virus belonging to this more diverse branch.

### 4.3. The Genetic Evolution of PIV5

In agreement with the previous study [6], the F and HN genes of PIV5 were observed under purifying selection, as the overall dN/dS values (0.209 and 0.154, respectively) were less than one. However, by using the M8 model, which proportioned the codons into different site classes (conserved, completely neutral, and positively selected), this study revealed that some codons in both the F and HN genes had elevated dN/dS values and were larger than one (Figure 5). Those codons were predicted to have been under a greater selection pressure than the rest. On the evolutionary path of the F and HN phylogenies (Figure 6 and Figure 7), most of those codons (6/9 for the F gene, 7/8 for the HN gene) were loaded on internal branches. Generally, substitutions occurring in internal branches were acknowledged to provide some beneficial traits for viruses [38].

Compared to the previous study [6], this study with a larger number of sequences being analyzed shed light on the diversity of PIV5 based on phylogenetic classification and the natural selection operated on the HN and F genes. The footprints of natural selection might be obscured with a limited number of sequences for genetic analysis. For example, with 16 PIV5 sequences, the previous study detected variation in only 1/5 codons (342), promoting resistance to neutralizing antibodies [6]. It was noteworthy that this study showed five codons of the HN gene (342, 437, 457, 491, 536) that are linked to resistance against neutralizing antibodies [9] were all located on internal branches, especially those leading to the more diverse group 2.2.2 (Figure 7), and there were 3/5 codons (342, 457, 536) that still might be under diversifying selection (dN/dS > 1, Figure 7). For PIV5, up to date, most of the experimental studies focused on the roles of the F and HN proteins in the membrane fusion process [26,27,28,29,39]. There are no available experiments to study the role(s) of immune-driven selection of the entire predicted codons above (Figure 5). In spite of that, this study made a contribution by elucidating the evolution path of the F and HN genes of PIV5.

## 5. Conclusions

This study confirmed the prevalence of PIV5 at the average rate of 1.78% in pigs in Republic of Korea for three consecutive years. The PIV5 circulating in Korean pigs belonged to two groups, of which group 2.2.2 was the most diverse. The evolution of two structural protein-coding genes (F and HN) were largely under purifying selection. A few codons (six for the F gene, seven for the HN gene) had elevated dN/dS values, which were loaded on internal branches and were predicted to relate to beneficial trait(s) of the virus.

## Figures and Tables

**Table 1:** Summary of the number of samples used to screen for PIV5.

Type of Samples	Number of Samples According to Year of Collection		
	2016	2017	2018
Serum	2142	1413	1035
Pooled organs	489	304	183

## Data Availability

The data supporting the findings of this study are included in the Supplementary Materials.

## Supplementary Materials

The following supporting information can be downloaded at: https://www.mdpi.com/article/10.3390/vetsci10070414/s1, Table S1: List of primers; Table S2: List of sequences, Figure S1: Phylogenetic-F; Figure S2: Phylogenetic-HN; Figure S3: Ancestral-reconstruction-F; Figure S4: Ancestral-reconstruction-HN.
