# Supplementary material for: PCR-Based Detection and Genetic Characterization of Parainfluenza Virus 5 Detected in Pigs in Korea from 2016 to 2018
Source: Vet Sci. 2023 Jun 25;10(7):414. doi: 10.3390/vetsci10070414 (PMC10384901; doi:10.3390/vetsci10070414)

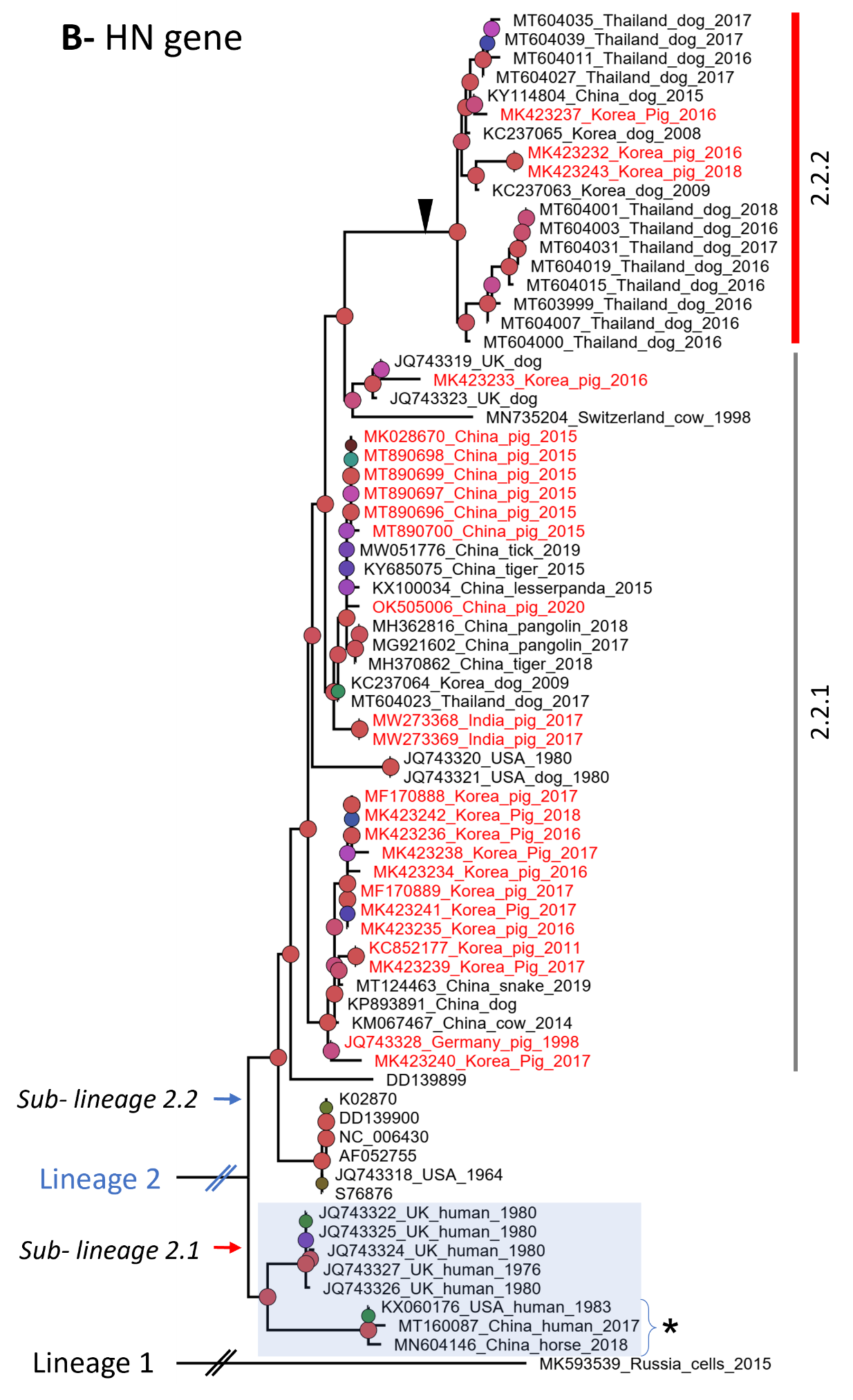


**Supplementary Figure S2.** The maximum likelihood phylogenetic tree of PIV5 based on the structure HN gene


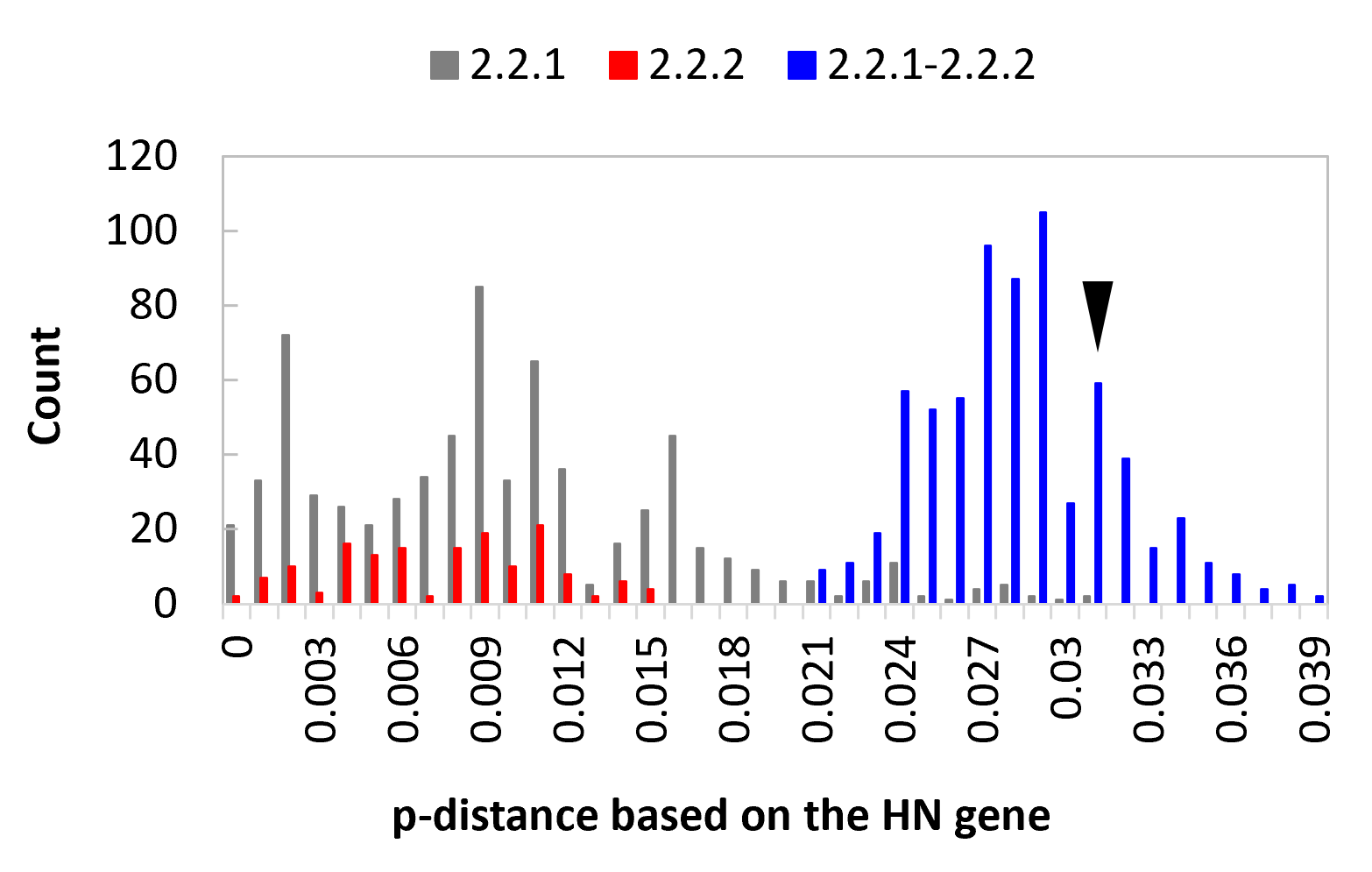

Supplement: Supplementary file 1 [file vetsci-10-00414-s001.zip › Supplementary-Figure-S2-phylogenetic-HN.docx]
