# Supplementary material for: PCR-Based Detection and Genetic Characterization of Parainfluenza Virus 5 Detected in Pigs in Korea from 2016 to 2018
Source: Vet Sci. 2023 Jun 25;10(7):414. doi: 10.3390/vetsci10070414 (PMC10384901; doi:10.3390/vetsci10070414)
Supplement: Supplementary file 1 [file vetsci-10-00414-s001.zip › Supplementary-Table-S1-list of primers.docx]

**Supplementary Table S1.** List of primers used for PIV5 detection and genome sequencing

| **Primer** | **Sequence (5’-3’)** | **Position** | **Purposes** | **Expected  size (bp)** |
| --- | --- | --- | --- | --- |
| PIV-F | CTATGCACACTACTAGCATTAAGCAT | 6653- 6954 | Detection | 302 |
| PIV-R | ATGTATCTATTATTATTAATCAGTGCAG |  |  |  |
| 1F | ACCAGGGGGAAAATGAAGTGGTG | 1-1200 | Genome Sequencing | 1200 |
| 1R | TAGTTCCTCATGTTGACATCTAACA |  |  |  |
| 2F | ACTTGCTTTAGCTGCATTTTCAG | 1000-2201 | Genome Sequencing | 1202 |
| 2R | TTAGATCAAGCACAGTTTGGGTGC |  |  |  |
| 3F | CTAACCAATGCTGCAGAGGC | 2000-3227 | Genome Sequencing | 1228 |
| 3R | ACCCCCATCACTGTTGATCACA |  |  |  |
| 4F | TCAAGAAAGCAATCATCCGCAGTG | 2995-4204 | Genome Sequencing | 1210 |
| 4R | CGGCGATGCTTAGATGAAATTTT |  |  |  |
| 5F | GGAAAGGGTGGATGGAGCTG | 4020-5206 | Genome Sequencing | 1187 |
| 5R | ATCCTTAAAGCTTGAATTGTAATAGG |  |  |  |
| 6F | CACACAATCACTAGGAACGGC | 5000-6242 | Genome Sequencing | 1243 |
| 6R | CAATACTAGTTTCGGTCCTATCG |  |  |  |
| 7F | TCTGTCTTGGATCGTTAGGTTTAAT | 6001-7237 | Genome Sequencing | 1237 |
| 7R | GAGATATAGAGTCTTTAGGGTTC |  |  |  |
| 8F | TCGCAATCTGACACTTGGCCC | 6994-8226 | Genome Sequencing | 1233 |
| 8R | GATGAGGACAATTCAATAATATAGATA |  |  |  |
| 9F | TACTGACCAACCCTTCGTCTACCAG | 7986-9222 | Genome Sequencing | 1236 |
| 9R | TATGTTATTCTTTTCTTCAGAGGATT |  |  |  |
| 10F | AAACAGATCATAATGATCCTGATTTAATC | 8994-10219 | Genome Sequencing | 1226 |
| 10R | TTTTGTTAATGATAGCTGATTCATCAC |  |  |  |
| 11F | CAAATTCGACCCGAATGTGGA | 9979-11249 | Genome Sequencing | 1271 |
| 11R | TGCATGACAGGTAGTTTAGACCTC |  |  |  |
| 12F | GTCCTAGGAGAATGCACCCAATC | 10988-12218 | Genome Sequencing | 1231 |
| 12R | TGTGAGACAAATCCAGTGCATC |  |  |  |
| 13F | GACAATCGATTCACATGGTTTTTCT | 11975-13219 | Genome Sequencing | 1245 |
| 13R | GAGAATAAGGCTATCTTCAGATGTC |  |  |  |
| 14F | GGTGCAGCATTAAACAATCTGGC | 12992-14219 | Genome Sequencing | 1228 |
| 14R | TTGTAGCAGTGATGATAGCATTAATTA |  |  |  |
| 15F | TGGCAAGCACACACAGATCAATAT | 13991-15246 | Genome Sequencing | 1256 |
| 15R | ACCAAGGGGAAAACTAAGATTAAT |  |  |  |
