# Supplementary material for: PCR-Based Detection and Genetic Characterization of Parainfluenza Virus 5 Detected in Pigs in Korea from 2016 to 2018
Source: Vet Sci. 2023 Jun 25;10(7):414. doi: 10.3390/vetsci10070414 (PMC10384901; doi:10.3390/vetsci10070414)
Supplement: Supplementary file 1 [file vetsci-10-00414-s001.zip › Supplementary-Table-S2-list of sequences.docx]

**Supplementary Table S2.** Information of sequences used in this study

| **No.** | **GenBank** | **Country** | **Host** | **Year  of isolation** | **Complete genome** | **F  gene** | **HN  gene** | **Used for p-distance calculation** |
| --- | --- | --- | --- | --- | --- | --- | --- | --- |
| 1 | MW273368 | India | pig | 2017 | ✓ | ✓ | ✓ | ✓ |
| 2 | MW273369 | India | pig | 2017 | ✓ | ✓ | ✓ | ✓ |
| 3 | MK593539 | Russia | cells | 2015 | ✓ | ✓ | ✓ |  |
| 4 | MT890696 | China | pig | 2015 | ✓ | ✓ | ✓ | ✓ |
| 5 | MT890699 | China | pig | 2015 | ✓ | ✓ | ✓ | ✓ |
| 6 | MT890700 | China | pig | 2015 | ✓ | ✓ | ✓ | ✓ |
| 7 | MT890698 | China | pig | 2015 | ✓ | ✓ | ✓ | ✓ |
| 8 | MT890697 | China | pig | 2015 | ✓ | ✓ | ✓ | ✓ |
| 9 | MK028670 | China | pig | 2015 | ✓ | ✓ | ✓ | ✓ |
| 10 | MW051776 | China | tick | 2019 | ✓ | ✓ | ✓ | ✓ |
| 11 | MG921602 | China | pangolin | 2017 | ✓ | ✓ | ✓ | ✓ |
| 12 | MH362816 | China | pangolin | 2018 | ✓ | ✓ | ✓ | ✓ |
| 13 | MH370862 | China | tiger | 2018 | ✓ | ✓ | ✓ | ✓ |
| 14 | KY685075 | China | tiger | 2015 | ✓ | ✓ | ✓ | ✓ |
| 15 | KX100034 | China | lesser panda | 2015 | ✓ | ✓ | ✓ | ✓ |
| 16 | KC237064 | Korea | dog | 2009 | ✓ | ✓ | ✓ | ✓ |
| 17 | MK423243 | Korea | pig | 2018 | ✓ | ✓ | ✓ | ✓ |
| 18 | MK423232 | Korea | pig | 2016 | ✓ | ✓ | ✓ | ✓ |
| 19 | KC237063 | Korea | dog | 2009 | ✓ | ✓ | ✓ | ✓ |
| 20 | MK423237 | Korea | pig | 2016 | ✓ | ✓ | ✓ | ✓ |
| 21 | KY114804 | China | dog | 2015 | ✓ | ✓ | ✓ | ✓ |
| 22 | KC237065 | Korea | dog | 2008 | ✓ | ✓ | ✓ | ✓ |
| 23 | MK423233 | Korea | pig | 2016 | ✓ | ✓ | ✓ | ✓ |
| 24 | JQ743319 | UK | dog |  | ✓ | ✓ | ✓ | ✓ |
| 25 | JQ743323 | UK | dog |  | ✓ | ✓ | ✓ | ✓ |
| 26 | MN735204 | Switzerland | cow | 1998 | ✓ | ✓ | ✓ | ✓ |
| 27 | MK423242 | Korea | pig | 2018 | ✓ | ✓ | ✓ | ✓ |
| 28 | MK423236 | Korea | pig | 2016 | ✓ | ✓ | ✓ | ✓ |
| 29 | MF170888 | Korea | pig | 2017 | ✓ | ✓ | ✓ | ✓ |
| 30 | MK423238 | Korea | pig | 2017 | ✓ | ✓ | ✓ | ✓ |
| 31 | MK423241 | Korea | pig | 2017 | ✓ | ✓ | ✓ | ✓ |
| 32 | MF170889 | Korea | pig | 2017 | ✓ | ✓ | ✓ | ✓ |
| 33 | MK423235 | Korea | pig | 2016 | ✓ | ✓ | ✓ | ✓ |
| 34 | MK423234 | Korea | pig | 2016 | ✓ | ✓ | ✓ | ✓ |
| 35 | MK423239 | Korea | Pig | 2017 | ✓ | ✓ | ✓ | ✓ |
| 36 | KC852177 | Korea | pig | 2011 | ✓ | ✓ | ✓ | ✓ |
| 37 | MT124463 | China | snake | 2019 | ✓ | ✓ | ✓ | ✓ |
| 38 | KM067467 | China | cow | 2014 | ✓ | ✓ | ✓ | ✓ |
| 39 | KP893891 | China | dog |  | ✓ | ✓ | ✓ | ✓ |
| 40 | JQ743328 | Germany | pig | 1998 | ✓ | ✓ | ✓ | ✓ |
| 41 | MK423240 | Korea | pig | 2017 | ✓ | ✓ | ✓ | ✓ |
| 42 | JQ743321 | USA | dog | 1980 | ✓ | ✓ | ✓ | ✓ |
| 43 | JQ743320 | USA |  | 1980 | ✓ | ✓ | ✓ | ✓ |
| 44 | DD139899 |  |  |  | ✓ | ✓ | ✓ |  |
| 45 | DD139900 |  |  |  | ✓ | ✓ | ✓ |  |
| 46 | NC_006430 |  |  |  | ✓ | ✓ | ✓ |  |
| 47 | JQ743318 | USA |  | 1964 | ✓ | ✓ | ✓ |  |
| 48 | JQ743326 | UK | human | 1980 | ✓ | ✓ | ✓ |  |
| 49 | JQ743325 | UK | human | 1980 | ✓ | ✓ | ✓ |  |
| 50 | JQ743322 | UK | human | 1980 | ✓ | ✓ | ✓ |  |
| 51 | JQ743327 | UK | human | 1976 | ✓ | ✓ | ✓ |  |
| 52 | JQ743324 | UK | human | 1980 | ✓ | ✓ | ✓ |  |
| 53 | MT160087 | China | human | 2017 | ✓ | ✓ | ✓ |  |
| 54 | KX060176 | USA | human | 1983 | ✓ | ✓ | ✓ |  |
| 55 | MN604146 | China | horse | 2018 | ✓ | ✓ | ✓ |  |
| 56 | KX808602 | China | tiger |  |  | ✓ |  | ✓ |
| 57 | AF052755 |  |  |  |  | ✓ | ✓ |  |
| 58 | AB021962 |  |  |  |  | ✓ |  |  |
| 59 | DD139915 |  |  |  |  | ✓ |  |  |
| 60 | AX586953 |  |  |  |  | ✓ |  |  |
| 61 | AB033629 |  |  |  |  | ✓ |  | ✓ |
| 62 | MT603999 | Thailand | dog | 2016 |  | ✓ | ✓ | ✓ |
| 63 | MT604006 | Thailand | dog | 2016 |  | ✓ |  | ✓ |
| 64 | MT604001 | Thailand | dog | 2018 |  | ✓ | ✓ | ✓ |
| 65 | MT604002 | Thailand | dog | 2016 |  | ✓ |  | ✓ |
| 66 | MT604018 | Thailand | dog | 2016 |  | ✓ |  | ✓ |
| 67 | MT604030 | Thailand | dog | 2017 |  | ✓ |  | ✓ |
| 68 | MT604010 | Thailand | dog | 2016 |  | ✓ |  | ✓ |
| 69 | MT604026 | Thailand | dog | 2017 |  | ✓ |  | ✓ |
| 70 | MT604034 | Thailand | dog | 2017 |  | ✓ |  | ✓ |
| 71 | MT604038 | Thailand | dog | 2017 |  | ✓ |  | ✓ |
| 72 | MT604000 | Thailand | dog | 2016 |  | ✓ | ✓ | ✓ |
| 73 | AJ749990 | UK | dog |  |  | ✓ |  | ✓ |
| 74 | AJ749991 | UK | dog |  |  | ✓ |  | ✓ |
| 75 | OK505006 | China | pig | 2020 |  | ✓ | ✓ | ✓ |
| 76 | OP264078 | China | dog | 2015 |  | ✓ |  | ✓ |
| 77 | KY364869 | China | pig | 2015 |  | ✓ |  | ✓ |
| 78 | KX808601 | China | tiger |  |  | ✓ |  | ✓ |
| 79 | KY364871 | China | pig | 2015 |  | ✓ |  | ✓ |
| 80 | KY364872 | China | pig | 2015 |  | ✓ |  | ✓ |
| 81 | KY364870 | China | pig | 2015 |  | ✓ |  | ✓ |
| 82 | KY364873 | China | pig | 2015 |  | ✓ |  | ✓ |
| 83 | MT604014 | Thailand | dog | 2016 |  | ✓ |  | ✓ |
| 84 | MT604022 | Thailand | dog | 2017 |  | ✓ |  | ✓ |
| 85 | AJ749980 | Germany | dog |  |  | ✓ |  | ✓ |
| 86 | AJ749992 | Germany | dog |  |  | ✓ |  | ✓ |
| 87 | AJ749986 | UK | human |  |  | ✓ |  |  |
| 88 | AJ749989 | UK | human |  |  | ✓ |  |  |
| 89 | AJ749988 | UK | human |  |  | ✓ |  |  |
| 90 | AJ749987 | UK | human |  |  | ✓ |  |  |
| 91 | MK117734 | China | horse | 2018 |  | ✓ |  |  |
| 92 | MK117736 | China | horse | 2017 |  | ✓ |  |  |
| 93 | MK117735 | China | horse | 2017 |  | ✓ |  |  |
| 94 | DD139914 |  |  |  |  | ✓ |  |  |
| 95 | K02253 |  |  |  |  | ✓ |  |  |
| 96 | AX586951 |  |  |  |  | ✓ |  |  |
| 97 | MT604011 | Thailand | dog | 2016 |  |  | ✓ | ✓ |
| 98 | MT604035 | Thailand | dog | 2017 |  |  | ✓ | ✓ |
| 99 | MT604039 | Thailand | dog | 2017 |  |  | ✓ | ✓ |
| 100 | MT604027 | Thailand | dog | 2017 |  |  | ✓ | ✓ |
| 101 | MT604003 | Thailand | dog | 2016 |  |  | ✓ | ✓ |
| 102 | MT604031 | Thailand | dog | 2017 |  |  | ✓ | ✓ |
| 103 | MT604019 | Thailand | dog | 2016 |  |  | ✓ | ✓ |
| 104 | MT604015 | Thailand | dog | 2016 |  |  | ✓ | ✓ |
| 105 | MT604007 | Thailand | dog | 2016 |  |  | ✓ | ✓ |
| 106 | MT604023 | Thailand | dog | 2017 |  |  | ✓ | ✓ |
| 107 | S76876 |  |  |  |  |  | ✓ |  |
| 108 | K02870 |  |  |  |  |  | ✓ |  |

*Note: empty cells indicated no available information*
