# Supplementary figures and images for: PCR-Based Detection and Genetic Characterization of Parainfluenza Virus 5 Detected in Pigs in Korea from 2016 to 2018
Source: Vet Sci. 2023 Jun 25;10(7):414. doi: 10.3390/vetsci10070414 (PMC10384901; doi:10.3390/vetsci10070414)

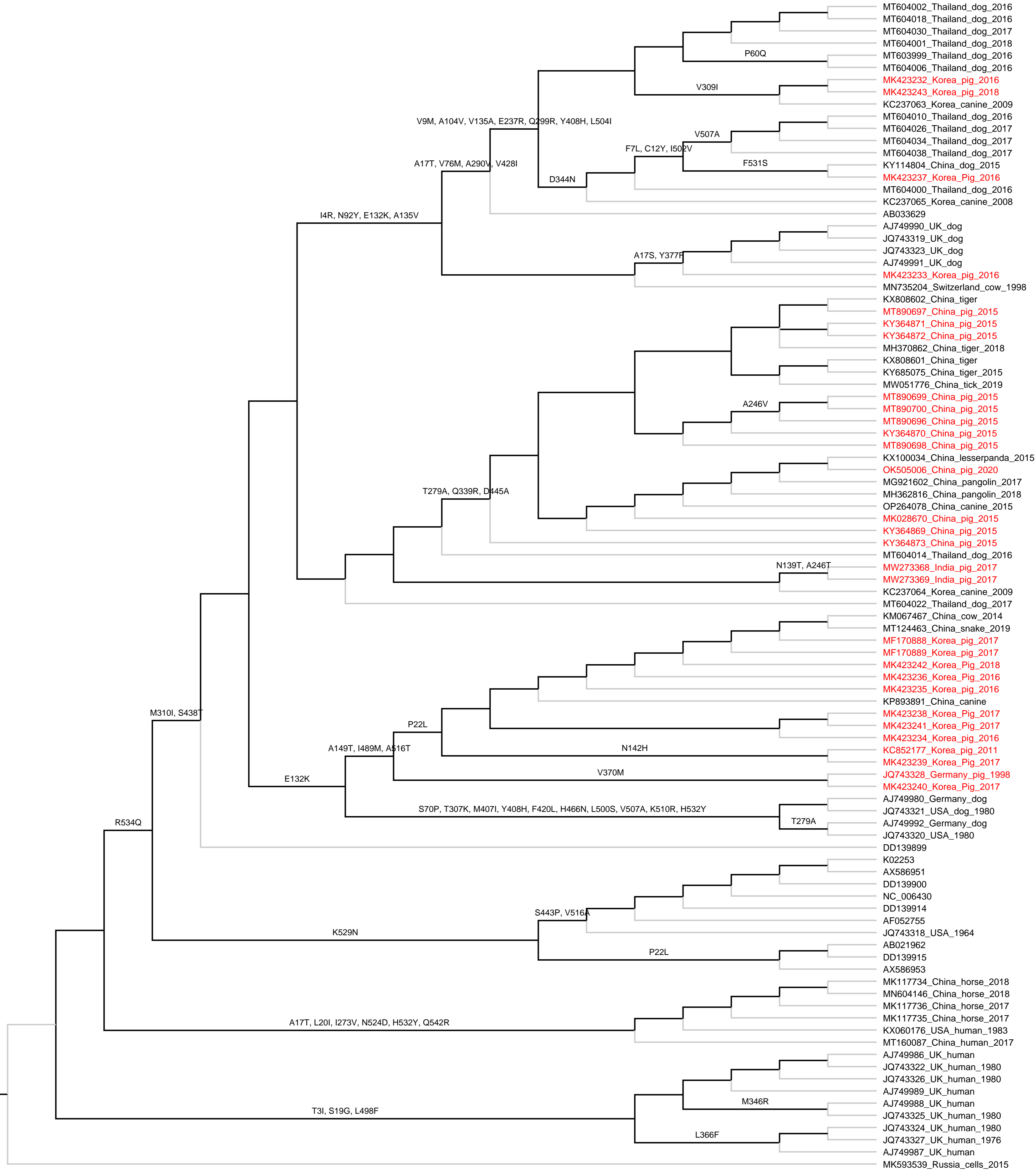

Supplement: Supplementary file 1 [file vetsci-10-00414-s001.zip › Supplementary-Figure-S3-ancestral-reconstruction-F.pdf]

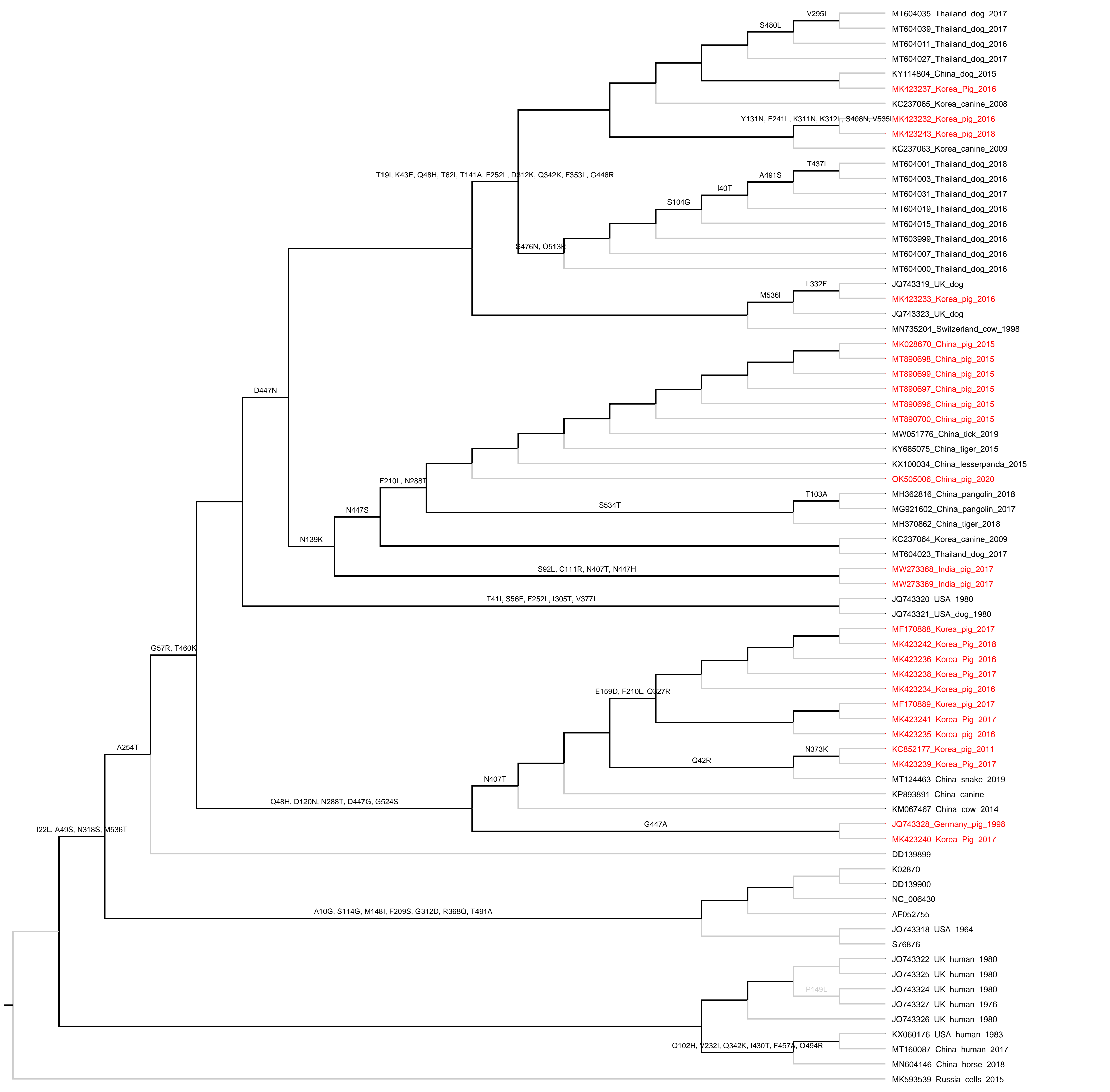

Supplement: Supplementary file 1 [file vetsci-10-00414-s001.zip › Supplementary-Figure-S4-ancestral-reconstruction-HN.pdf]
